# Clinical characteristics and prognosis of immunoglobulin light chain amyloidosis patients

**DOI:** 10.3389/fmed.2026.1737636

**Published:** 2026-01-29

**Authors:** Lijun Song, Yan Su, Rong Li, Siyi Du, Yimiao Xue, Yihai Gu, Junmin Chen, Xing Xing, Ziyuan Shen, Yuping Wei

**Affiliations:** 1 Department of Hematology, People's Hospital of Ningxia Hui Autonomous Region, Ningxia Medical University, Yinchuan, Ningxia, China; 2 Department of Biostatistics, Johns Hopkins Bloomberg School of Public Health, Baltimore, MD, United States; 3 Clinical Research Institute, The Affiliated Hospital of Xuzhou Medical University, Xuzhou, Jiangsu, China

**Keywords:** AL amyloidosis, BMPCs, fibrinogen degradation products, prognosis, risk stratification

## Abstract

**Objective:**

To analyze the prognostic factors associated with overall survival (OS) in patients diagnosed with immunoglobulin light chain (AL) amyloidosis, with the goal of improving risk stratification and patient management.

**Methods:**

A retrospective cohort analysis was conducted on 87 patients diagnosed with AL amyloidosis at the People’s Hospital of Ningxia Hui Autonomous Region from January 2016 to December 2023. Demographic, clinical, laboratory data, and survival status were collected. Univariable and multivariable Cox regression analyses were used to identify significant predictors of OS. Kaplan–Meier survival curves and restricted cubic spline (RCS) analysis were employed to evaluate risk stratification.

**Results:**

The median overall survival was 22.0 months (95% CI: 15.2–28.8), and the median follow-up duration was 39.0 months (95% CI: 29.2–48.8). Our results revealed that higher bone marrow plasma cell count (BMPCs), myeloma presence, heart failure, and increased fibrinogen degradation products (FDP) were significantly associated with poor survival outcomes. The median survival time for patients with BMPCs >12% was 13 months, compared to 36 months for those with lower BMPCs. Myeloma presence was the strongest predictor of survival, with a median survival of 15 months for those with myeloma versus 36 months for those without. Multivariable analysis identified that myeloma (*HR* = 2.582, 95% CI: 1.105–6.034, *p* = 0.029), heart failure (*HR* = 2.258, 95% CI: 1.098–4.641, *p* = 0.027), higher BMPCs (*HR* = 1.018, 95% CI: 1.001–1.035, *p* = 0.035), and elevated FDP levels (*HR* = 1.018, 95% CI: 1.004–1.017, *p* = 0.001) were independent risk factors for death.

**Conclusion:**

Elevated BMPCs, concurrent myeloma, heart failure, and increased FDP levels were associated with poor OS of AL amyloidosis patients. These factors could be incorporated into clinical decision-making to better stratify risk and guide treatment strategies for AL amyloidosis patients.

## Introduction

Immunoglobulin light chain (AL) amyloidosis is a systemic disease characterized by the deposition of *β*-pleated sheets of monoclonal immunoglobulin light chains, predominantly of the *κ* or *λ* types, which are secreted by clonal populations of bone marrow plasma cells (1). This condition may occur alongside any immunoglobulin-secreting B-cell neoplasm. Unlike most proteins, which conform to an *α*-helical structure, the light chain proteins in AL amyloidosis misfold, forming *β*-pleated sheets that result in multi-organ damage (2). The rarity and complexity of AL amyloidosis pose significant challenges in both diagnosis and treatment, largely due to the limited availability of comprehensive, detailed patient data.

Although therapeutic advances, including proteasome inhibitors, immunomodulatory agents, and monoclonal antibodies such as daratumumab, have improved hematologic responses and organ function in selected patients, the overall prognosis for AL amyloidosis remains unsatisfactory for many (3–5). In particular, cardiac involvement continues to be a major determinant of survival, with advanced cardiac amyloidosis associated with markedly worse outcomes (6, 7). However, even among patients with similar cardiac or hematologic status, survival can vary significantly, suggesting that current prognostic models may not fully capture disease heterogeneity.

To address this gap, we evaluated a range of routinely available clinical and laboratory parameters that are commonly assessed at the time of diagnosis. While variables such as bone marrow plasma cell percentage and cardiac involvement have been individually studied in previous reports (8, 9), others have received limited attention in the context of AL amyloidosis. By re-examining these indicators, this study seeks to explore their prognostic relevance and provide additional insights to support individualized risk stratification. Therefore, this study aimed to evaluate prognostic factors influencing overall survival in patients with AL amyloidosis.

## Materials and methods

### Study design

We retrospectively retrieved data from 87 patients diagnosed with AL amyloidosis at the People’s Hospital of Ningxia Hui Autonomous Region from January 2016 to December 2023. The diagnosis of AL amyloidosis and assessment of organ involvement were based on the consensus criteria modified in 2021 (10). Patients with hereditary amyloidosis, AA amyloidosis, or other non-AL types of systemic amyloidosis were excluded. Ethics approval was obtained from the People’s Hospital of Ningxia Hui Autonomous Region (NZR-107), and the study adhered to the principles outlined in the Declaration of Helsinki.

### Covariates

The following variables were collected (11–15): age, gender, lactate dehydrogenase (LDH), white blood cell count (WBC), lymphocyte count (LYC), monocyte (MONO), thrombin time (TT), fibrinogen degradation products (FDP), neutrophils (NE), hemoglobin (HGB), erythrocyte sedimentation rate (ESR), β2-microglobulin (β2-MG), aspartate aminotransferase (AST), bone marrow plasma cells (BMPCs), and activated partial thromboplastin time (APTT). Heart failure was defined according to the 2021 European Society of Cardiology (ESC) Guidelines (16), as a documented clinical diagnosis supported by echocardiographic evidence of cardiac dysfunction (e.g., reduced left ventricular ejection fraction <50%) and/or typical symptoms such as dyspnea or fatigue. All laboratory parameters were measured in the Clinical Laboratory Department of the People’s Hospital of Ningxia Hui Autonomous Region using certified automated analyzers and standard operating procedures. Specifically, β2-MG and LDH were measured using the Beckman Coulter Remisol integrated system, FDP were assessed using the Werfen ACL TOP 700 coagulation analyzer, and WBC and HGB were analyzed using the Mindray CAL8000 automated hematology platform. Other routine hematologic and biochemical indicators were measured using the hospital’s standardized laboratory systems with regular internal quality control.

### Follow-up and endpoints

Follow-up data were collected through a review of inpatient medical records and telephone interviews. The follow-up period concluded in October 2024. Overall survival (OS) was defined as the time interval between the date of diagnosis and either death or the last follow-up. The survival status of all patients was verified through death records or telephone communication, either with the next of kin in cases of deceased patients or with the patients themselves.

### Statistical analysis

Continuous variables were expressed with median (1st quartile and 3rd quartile) values or mean (S. D.) and analyzed by Student’s *t*-test or Mann–Whitney U test according to the result of the Shapiro–Wilk normality test. We used categorical variables that had frequency and corresponding percentage values. Fisher’s exact and chi-square tests were used for between-group comparisons. To determine the prognostic factors that affect overall survival time, we first use univariable Cox proportional hazard regression analysis. Results were reported as hazard ratios (HR) with 95% confidence intervals (CI). Then, a backward selection procedure for variables with *p* < 0.1 for univariable regression was applied to determine the strongest predictors for survival and independent risk factors. Moreover, optimal cutoff values for continuous variables among strong predictors were determined for risk stratification based on restricted cubic spline (RCS) analysis. Risk stratification was visualized by the Kaplan–Meier method, and the log-rank test was used to compare survival distributions between groups. The data was statistically processed by R software (version 4.2.2). In all statistical analyses, *p* < 0.05 was defined as statistically significant.

## Results

### Baseline clinical characteristics

A total of 87 AL amyloidosis patients were included in the final analysis (Table 1). The mean age of patients was 63 years, and the majority (59.8%) were male. As for the clinical phenotype, 42.5% presented with myeloma, 67.8% with CRAB, 32.9% with renal involvement, and 24.7% with heart failure. The study cohort was divided into 2 groups depending on the prognosis: death (*n* = 44) and survivors (*n* = 43). Comparing the baseline demographic and experimental characteristics, patients in these 2 groups were not significantly different in sex or LDH, but in age, myeloma, BMPCs, hemoglobin, and the difference in heart failure.

**Table 1:** Clinical parameters of patients died and survive from AL amyloidosis.

Variables	Overall (*n* = 87)	Survivors (*n* = 43)	Death (*n* = 44)	*P*
Age (mean (SD), year)	63.00 (9.66)	60.67 (7.53)	65.27 (10.98)	0.026
Sex (%)				0.221
Male	52 (59.8)	29 (67.4)	23 (52.3)	
Female	35 (40.2)	14 (32.6)	21 (47.7)	
BMPCs (median [IQR])	8.00% [3.00, 27.00%]	6.00% [2.00, 9.50%]	18.50% [5.75, 37.00%]	<0.001
Myeloma (%)				<0.001
No	50 (57.5)	35 (81.4)	15 (34.1)	
Yes	37 (42.5)	8 (18.6)	29 (65.9)	
Heart failure				0.045
No	61 (75.3)	33 (86.8)	28 (65.1)	
Yes	20 (24.7)	5 (13.2)	15 (34.9)	
WBC {[median (IQR)], 10^9^/L}	5.33 [3.59, 7.52]	5.28 [3.66, 7.10]	5.34 [3.55, 7.80]	0.997
NE {[median (IQR)], 10^9^/L}	3.19 [2.08, 4.64]	3.19 [2.15, 3.91]	3.18 [2.01, 5.28]	0.911
HGB [mean (SD), g/L]	107.38 (30.85)	115.21 (29.65)	99.91 (30.43)	0.021
PLT {[median (IQR)], 10^9^/L}	174.00 [109.00, 267.00]	186.00 [133.00, 268.00]	163.50 [101.50, 257.00]	0.463
ESR {[median (IQR)], mm/h}	42.00 [16.50, 70.25]	40.00 [15.00, 65.00]	44.50 [18.25, 71.00]	0.693
LDH {[median (IQR)], U/L}	203.00 [159.00, 246.50]	194.00 [151.00, 230.00]	209.50 [165.00, 250.50]	0.208
β2-MG {[median (IQR)], mg/L}	4.37 [2.78, 8.41]	3.01 [2.45, 6.19]	6.23 [3.61, 10.04]	0.001
FDP [mean (SD), μg/mL]	7.96 (38.81)	2.96 (3.39)	12.48 (53.36)	0.276
Treatment				0.001
Dara-based	8 (9.20)	8 (100)	0 (0)	
Vrd-based	58 (66.67)	20 (34.48)	38 (65.52)	

Values are presented as mean ± standard deviation (SD) for normally distributed continuous variables and as median (interquartile range) for non-normally distributed variables. Categorical variables are presented as number (percentage). *p*-values were calculated using Student’s *t*-test for normally distributed continuous variables, Mann–Whitney U test for non-normally distributed variables, and chi-square or Fisher’s exact test for categorical variables, as appropriate. A two-sided *P* < 0.05 was considered statistically significant. WBC, white blood cell; NE, neutrophils; HGB, hemoglobin; PLT, platelet; ESR, erythrocyte sedimentation rate; LDH, lactate dehydrogenase; β2-MG, β2-microglobulin; FDP, fibrinogen degradation products; BMPCs, bone marrow plasma cells.

**Figure 1:**
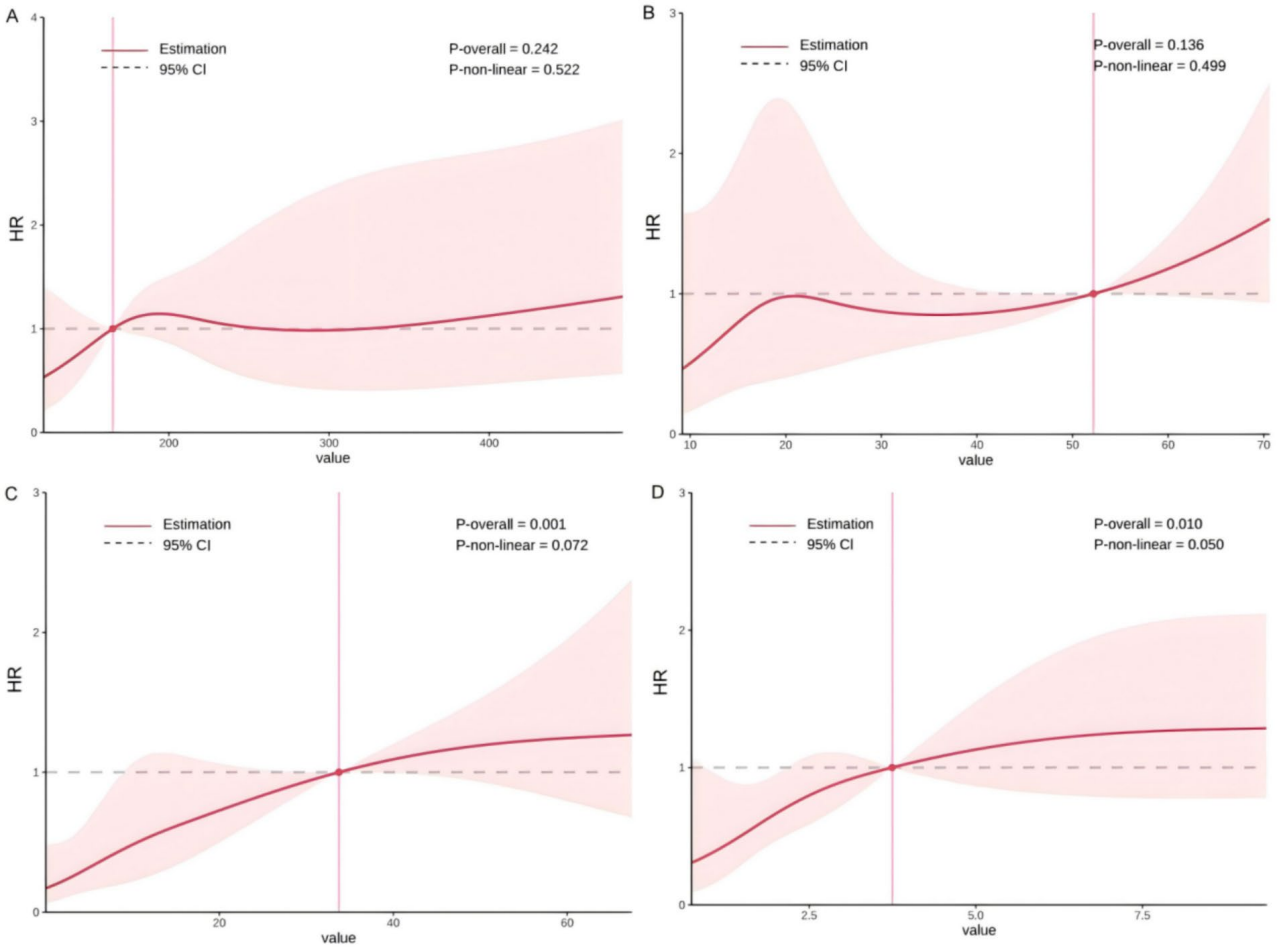
Restricted cubic spline analysis of continuous predictors and hazard ratio. **(A)** Lactic dehydrogenase; **(B)** aspartate aminotransferase; **(C)** bone marrow plasma cells; **(D)** fibrinogen degradation products.

**Figure 2:**
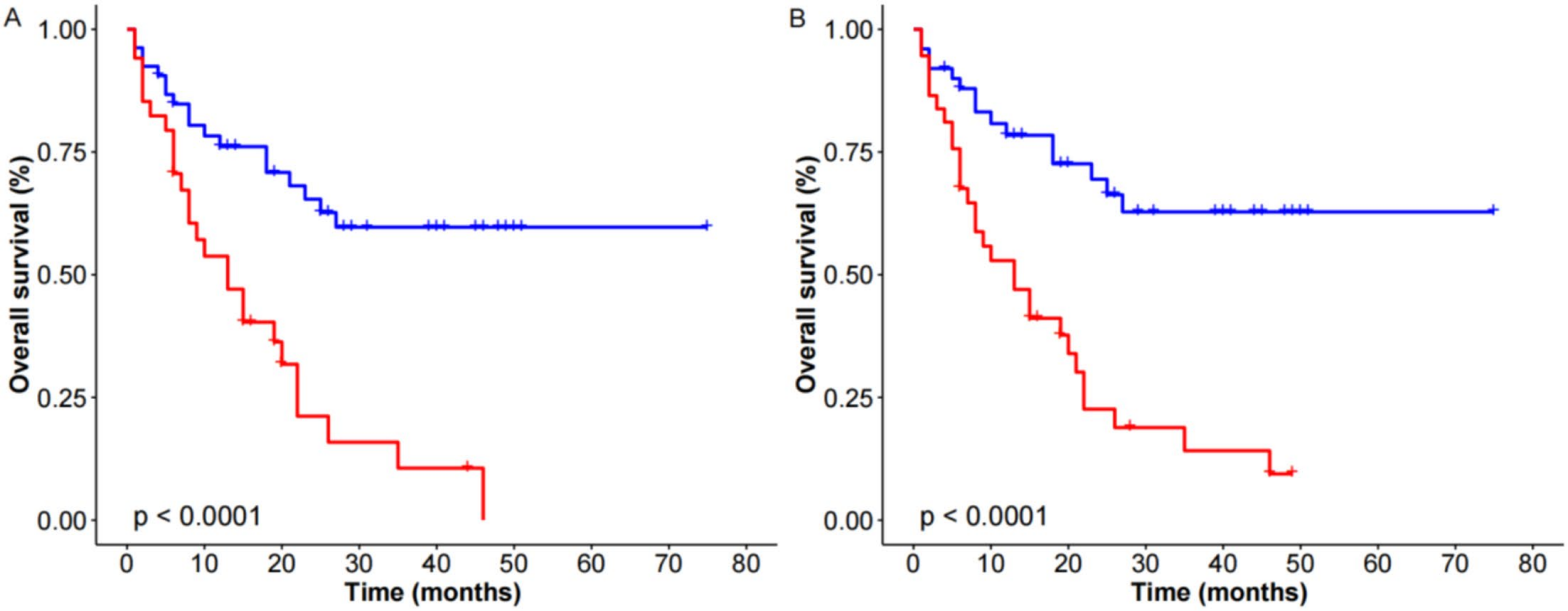
Kaplan–Meier survival curves stratified by strong factors, **(A)** bone marrow plasma cells and **(B)** myeloma.

Among the 87 patients, conventional karyotyping results were available for 68 individuals. A normal karyotype was observed in 90.3% (28/31) of survivors and 78.4% (29/37) of deceased patients, with no significant difference between groups (*p* = 0.20). Furthermore, molecular cytogenetic analysis was available in a subset of 28 patients. No significant differences in IGH translocations, 1q21 gain, TP53 deletion, or other common aberrations were observed between survivors and non-survivors (Figures 1, 2).

### Risk factors associated with death

In this study, a total of 44 (50.6%) patients died from the disease, with a mean time to death of 11.8 months. The median survival time was 22.0 (95% CI: 15.2–28.8) and the median follow-up time was 39.0 (95% CI: 29.2–48.8) months.

Table 2 displayed univariable analyses for the potential association between clinical parameters and death. The results showed that patients with myeloma, orthostatic hypotension, purpura, heart failure, higher BMPCs, lower hemoglobin, higher TT, higher FDP, higher D-dimer, higher serum free kappa light chain, and higher urine-involved light chain, higher differences between the involved light chain and the uninvolved light chain were correlated with increased risk of death. Furthermore, RCS analysis did not reveal a significant non-linear relationship between FDP and mortality risk (*P* for non-linearity ≥ 0.05).

**Table 2:** Univariable regression fitting the association between clinical parameters at presentation and death.

Variables	HR (95% CI)	*P*
Myeloma (yes)	3.653 (1.944–6.864)	<0.001
Orthostatic hypotension (yes)	2.765 (1.057–7.235)	0.038
Purpura (yes)	3.919 (1.804–8.516)	0.001
Heart failure (yes)	2.579 (1.372–4.845)	0.003
Age	1.035 (1.001–1.071)	0.044
BMPCs	1.021 (1.010–1.032)	<0.001
HGB	0.988 (0.978–0.999)	0.026
TT	1.117 (1.022–1.221)	0.015
FDP	1.008 (1.002–1.014)	0.009
D-Dimer	1.053 (1.016–1.091)	0.005
Serum free kappa light chain	1.000 (1.0001–1.0005)	0.004
Urine-involved light chain	1.002 (1.0003–1.0043)	0.023
Differences between the involved light chain and the uninvolved light chain	1.0003 (1.0001–1.0005)	0.003

Hazard ratios (HRs) and 95% confidence intervals (CIs) were estimated using univariable Cox proportional hazards models. Categorical variables were coded as binary (e.g., yes vs. no). A two-sided *P* < 0.05 was considered statistically significant. HGB, hemoglobin; TT, thrombin time; FDP, fibrinogen degradation products. Preliminary analyses using RCS were conducted to assess the linearity of the relationship between the continuous predictors and the hazard of the outcome. The Restricted cubic spline (RCS) analysis did not detect significant non-linear associations between the hazard ratio and continuous variables such as LDH, FDP, BMPCs, and AST (P for non-linearity ≥ 0.05).

The multivariable Cox model with variables had *p* < 0.1 for univariable analysis. Only myeloma, heart failure, higher BMPCs, higher AST, higher APTT, and higher FDP were independent risk factors for death (Table 3).

**Table 3:** Multivariable analysis fitting the association between clinical parameters at presentation and death.

Variables	HR (95% CI)	*P*
Myeloma (yes)	2.582 (1.105–6.034)	0.029
Orthostatic hypotension (yes)	2.396 (0.815–7.049)	0.112
Heart failure (yes)	2.258 (1.098–4.641)	0.027
BMPCs	1.018 (1.001–1.035)	0.035
AST	1.019 (1.004–1.033)	0.011
LDH	1.002 (1.000–1.003)	0.053
APTT	0.924 (0.854–0.998)	0.045
FDP	1.011 (1.004–1.017)	0.001

Results are expressed as hazard ratios (HRs) with 95% confidence intervals (CIs). A two-sided *P* < 0.05 was considered statistically significant. AST, Aspartate aminotransferase; LDH, lactic dehydrogenase; APTT, activated partial thromboplastin time; FDP, fibrinogen degradation products; BMPCs, bone marrow plasma cells.

### Risk stratifications

The optimal cutoffs for the continuous variables belonging to strong risk factors were determined for risk stratification (Table 3). According to the K-M curve, we found higher BMPCs >12%, and myeloma was the most effective risk stratification factor for the long-term clinical outcome. At 36 months, the survival rate for the group with higher BMPCs was 10.6% (95% CI: 3.01–37.15%), compared to 59.7% (95% CI: 46.47–76.65%) for the group with lower BMPCs. The median survival time for patients with BMPCs >12% was 13 months. At 36 months, the survival rate for patients with myeloma was 14.1% (95% CI: 5.50–36.3%), while it was 62% (95% CI: 49–80.5%) for those without myeloma. The median survival time for patients with myeloma was 15 months.

## Discussion

This study analyzed the clinical characteristics and prognostic factors of patients with AL amyloidosis. Our findings highlight several key factors influencing overall survival, including the presence of myeloma, heart failure, higher BMPCs, and specific biomarkers. The significant association between these variables and survival outcomes underscores the importance of early identification and tailored therapeutic strategies in AL amyloidosis management.

Our study indicated a significant association between higher BMPCs and myeloma with poorer survival outcomes in AL amyloidosis patients. This result highlights the potential of BMPCs as a key prognostic biomarker, as elevated BMPC levels could be indicative of more aggressive disease progression. Patients with myeloma had a significantly lower 36-month survival rate compared to those without, confirming its adverse prognostic effect. As a malignancy of plasma cells, myeloma accelerates AL amyloidosis progression by increasing amyloidogenic light chain production and enhancing organ damage (17, 18). Furthermore, the high burden of clonal plasma cells in the bone marrow has been linked to increased amyloid deposition, further exacerbating the progression of the disease (19–21). Our results are consistent with previous studies that demonstrated significantly worse outcomes in patients with concurrent multiple myeloma compared to those with AL amyloidosis alone (17, 22). Recent studies have also suggested that elevated BMPCs, even in the absence of overt multiple myeloma, contribute to disease progression in AL amyloidosis (20, 23). For instance, Tovar et al. (24). demonstrated that patients with BMPCs >10% had significantly higher levels of circulating free light chains and worse cardiac involvement. These findings support the biological relevance of BMPCs in the pathogenesis and progression of AL amyloidosis beyond their prognostic significance.

Our study also revealed a significant correlation between heart failure and increased mortality in AL amyloidosis patients. Heart failure, particularly in the setting of amyloid cardiomyopathy, is a common cause of death in these patients (25–27). This is consistent with previous findings (28–30), which demonstrated that cardiac involvement in AL amyloidosis is a major determinant of survival, with patients presenting with more severe cardiac involvement having significantly lower survival rates. Early detection of cardiac involvement using advanced imaging techniques such as echocardiography and cardiac MRI can aid in better prognostication and help clinicians intervene earlier, potentially reducing mortality rates associated with cardiac amyloidosis.

In this study, we also identified higher FDPs and longer APTT as independent risk factors for death in AL amyloidosis patients. Previous investigations have documented coagulation and fibrinolytic abnormalities in AL amyloidosis. Arahata et al. (31). reported that systemic AL amyloidosis patients often display abnormal coagulation and fibrinolysis features. Elevated levels of FDPs suggest that there is ongoing fibrinolysis within the body, which may be indicative of a systemic inflammatory response (32). In the context of AL amyloidosis, this heightened fibrinolytic activity is likely a consequence of amyloid deposits triggering a state of chronic inflammation and tissue damage. This chronic inflammation can, in turn, disrupt normal coagulation pathways, potentially increasing the risk of clotting and bleeding disorders (33, 34). Moreover, clotting alterations including prolonged APTT have been observed in AL amyloidosis patients, as evidenced by studies such as that by Gamba et al. (35), which found prolonged aPTT in a cohort of patients with primary systemic amyloidosis. Prolonged APTT could suggest a dysfunction in the coagulation cascade. In the case of AL amyloidosis, amyloid deposits in the vasculature and various organs might directly impair the normal function of coagulation factors or endothelial cells, leading to an increased risk of both hemorrhagic and thrombotic events. Amyloid fibrils can affect the vascular integrity, causing endothelial dysfunction, and might also interact with blood coagulation factors, resulting in a disrupted clotting process (36). These dysfunctions may exacerbate the already existing clinical challenges faced by AL amyloidosis patients, who are often dealing with multiple organ involvement and an unpredictable disease course (35, 37).

Taken together, these findings underscore the complexity of AL amyloidosis, as it is a multifaceted disease where not only the immune system and amyloid deposition drive organ failure, but the coagulation system is also intricately involved in the disease’s progression. The presence of dysregulated coagulation markers like elevated FDPs and prolonged APTT highlights the need for comprehensive management strategies that address not only the underlying plasma cell dyscrasia but also the associated coagulopathy. Monitoring coagulation parameters in AL amyloidosis patients may offer valuable insights into disease severity, help predict adverse outcomes, and guide clinicians in providing tailored treatments aimed at managing both the amyloidogenic process and the systemic effects of the disease, including its impact on coagulation. Moreover, these findings suggest that future therapeutic strategies may need to consider anticoagulation or antifibrinolytic therapies, particularly in high-risk patients with significant coagulopathy, to mitigate the adverse effects of amyloid-induced coagulopathy.

This study provides valuable insights into the clinical characteristics and prognostic factors of AL amyloidosis, emphasizing the importance of BMPCs, heart failure, and light chain differences as key predictors of survival. However, several limitations need to be acknowledged. First, the relatively small sample size may limit the generalizability of the findings, and larger, multicenter studies are needed to validate these results across a more diverse population. Second, due to the retrospective design and limited number of evaluable cases, treatment regimens were not included in the multivariable survival analysis. The heterogeneity in treatment approaches and the relatively early enrollment of many patients before the widespread use of daratumumab further limited our ability to assess treatment-specific effects. Future prospective studies with standardized treatment protocols will be essential to better elucidate the prognostic impact of therapeutic strategies. Third, quantitative cardiac biomarkers such as N-terminal pro-B-type natriuretic peptide and cardiac troponin I/T were not routinely available in our dataset. These biomarkers are central to the Mayo staging system, which is widely used for prognostic stratification in AL amyloidosis. Lastly, the absence of complete immunoglobulin heavy chain subtype data in our cohort prevented a detailed analysis of the associations between IgG subclasses and organ-specific involvement. Considering the potential relationship between immunoglobulin isotypes and the tropism of amyloid deposition, future multicenter studies incorporating standardized immunophenotyping are warranted to determine whether specific subtypes are linked to distinct patterns of organ damage. Despite these limitations, our findings highlight the need to refine current risk stratification models by incorporating additional biomarkers and accounting for disease heterogeneity. Further research is also warranted to investigate the role of emerging treatments such as daratumumab in high-risk populations to optimize patient outcomes.

## Data Availability

The raw data supporting the conclusions of this article will be made available by the authors, without undue reservation.
